# Changes in the proteomic and metabolic profiles of *Beta vulgaris *root tips in response to iron deficiency and resupply

**DOI:** 10.1186/1471-2229-10-120

**Published:** 2010-06-21

**Authors:** Rubén Rellán-Álvarez, Sofía Andaluz, Jorge Rodríguez-Celma, Gert Wohlgemuth, Graziano Zocchi, Ana Álvarez-Fernández, Oliver Fiehn, Ana Flor López-Millán, Javier Abadía

**Affiliations:** 1 Plant Nutrition Department, Aula Dei Experimental Station, CSIC, PO Box 13034, E-50080 Zaragoza, Spain; 2 Dipartimento di Produzione Vegetale, Sez. Biochimica e Fisiologia delle Piante, Università di Milano, Via Celoria 2, I-20133 Milano, Italy; 3 Genome Center, University of California Davis, CA 95616, USA; 4 Current address: Zeu-Inmunotec, C/Bari, n° 25, Duplicado, E-50197, Zaragoza, Spain

## Abstract

**Background:**

Plants grown under iron deficiency show different morphological, biochemical and physiological changes. These changes include, among others, the elicitation of different strategies to improve the acquisition of Fe from the rhizosphere, the adjustment of Fe homeostasis processes and a reorganization of carbohydrate metabolism. The application of modern techniques that allow the simultaneous and untargeted analysis of multiple proteins and metabolites can provide insight into multiple processes taking place in plants under Fe deficiency. The objective of this study was to characterize the changes induced in the root tip proteome and metabolome of sugar beet plants in response to Fe deficiency and resupply.

**Results:**

Root tip extract proteome maps were obtained by 2-D isoelectric focusing polyacrylamide gel electrophoresis, and approximately 140 spots were detected. Iron deficiency resulted in changes in the relative amounts of 61 polypeptides, and 22 of them were identified by mass spectrometry (MS). Metabolites in root tip extracts were analyzed by gas chromatography-MS, and more than 300 metabolites were resolved. Out of 77 identified metabolites, 26 changed significantly with Fe deficiency. Iron deficiency induced increases in the relative amounts of proteins and metabolites associated to glycolysis, tri-carboxylic acid cycle and anaerobic respiration, confirming previous studies. Furthermore, a protein not present in Fe-sufficient roots, dimethyl-8-ribityllumazine (DMRL) synthase, was present in high amounts in root tips from Fe-deficient sugar beet plants and gene transcript levels were higher in Fe-deficient root tips. Also, a marked increase in the relative amounts of the raffinose family of oligosaccharides (RFOs) was observed in Fe-deficient plants, and a further increase in these compounds occurred upon short term Fe resupply.

**Conclusions:**

The increases in DMRL synthase and in RFO sugars were the major changes induced by Fe deficiency and resupply in root tips of sugar beet plants. Flavin synthesis could be involved in Fe uptake, whereas RFO sugars could be involved in the alleviation of oxidative stress, C trafficking or cell signalling. Our data also confirm the increase in proteins and metabolites related to carbohydrate metabolism and TCA cycle pathways.

## Background

Two different strategies of Fe uptake have been described in plants. The so-called chelation strategy (or Strategy II), which is mainly found in graminaceous plants, is based on the excretion of phytosideropores (PS) to the rhizosphere. Phytosideropores rapidly chelate Fe(III), to form Fe(III)-PS chelates that are subsequently transported into the root cells through a specific transporter. The so-called reduction strategy (or Strategy I) relies on the coordinated action of a membrane bound Fe reductase, that reduces Fe(III) to Fe(II) [1], an Fe(II) uptake transporter [2] and an H^+ ^-ATPase that lowers the pH of the rhizosphere [3], is mainly used by non graminaceous plants, including *Beta vulgaris*. The reduction strategy includes root morphological, physiological and biochemical changes that lead to an increased capacity for Fe uptake. Morphological changes include root tip swelling, development of transfer cells and an increase in the number of lateral roots, leading to an increase in the root surface in contact with the medium [4].

Some plants are able to accumulate and/or release both reducing and chelating substances, such as phenolics and flavins, which may have a role in Fe acquisition [5,6]. Iron has been shown to down-regulate riboflavin (Rbfl) synthesis in flavinogenic yeast strains and some bacteria [7,8]. In plants, Rbfl and derivatives are accumulated and/or excreted in Fe-deficient roots and could act as a redox bridge for electron transport to the Fe(III) reductase [9]. Moreover, FRO2 belongs to a superfamily of flavocytochrome oxidoreductases [1], and a recent study confirmed that the FRO2 protein contains FAD sequence motifs on the inside of the membrane [10]. Also, a connection between Fe deficiency perception and Rbfl excretion has been described to occur through basic helix-loop-helix (bHLH) transcription factors in *Arabidopsis thaliana *[11].

At the metabolic level, increases in the activity of phospho*enol*pyruvate carboxylase (PEPC) and several enzymes of the glycolytic pathway and the tricarboxylic acid (TCA) cycle have been found in different plant species grown under Fe deficiency [12,13]. Transcriptomic [14] and proteomic studies in Fe deficient plants [15-17] have also reported increases in root transcript and protein abundances, respectively, of enzymes related to the glycolytic and TCA cycle pathways, among others. Iron deficiency also induces an accumulation of organic acids, mainly malate and citrate, in roots [12]. The induction of C metabolism in roots of Fe-deficient plants would not only provide a source of reducing power, protons and ATP for the Fe(III) reductase and H^+^-ATPase enzymes, but also lead to an anaplerotic root C fixation [18,19]. Carbon accumulated in roots is exported in the form of organic acids *via *xylem [18,20,21] to leaves [22], which have otherwise drastically reduced photosynthetic rates when Fe-deficient. The higher energy requirements in Fe-deficient root cells are tackled by increasing mitochondrial oxidative processes, and roots from Fe-deficient plants show enhanced respiratory activities and higher O_2 _consumption rates [9,23]. On the other hand, the mitochondrial respiratory chain is strongly affected under Fe-deficient conditions [23,24], since some of its components are Fe-containing enzymes. Iron deficiency leads to an enhancement of different ROS detoxification strategies [25]. Furthermore, an increase in anaerobic metabolism has also been described in Fe-deficient roots [9], probably as an strategy to oxidize all the reducing power generated by glycolysis and TCA cycle that can not be easily oxidized in the respiratory chain. When resupplied with Fe, Fe-deficient plants reorganize its metabolism by readjusting metabolic cycles and changing root morphology towards those typical of Fe-sufficient plants [21,26].

The most common approach used to study Fe deficiency in roots is to analyze only a small number of genes, proteins and/or metabolites. A more comprehensive knowledge of the processes taking place in Fe-deficient roots has been recently provided by the application of modern techniques allowing for the simultaneous and untargeted analysis of multiple genes or proteins [14-17]. The aim of this work was to characterize the changes induced in the root tip proteome and metabolome of sugar beet plants in response to Fe deficiency and resupply, in order to provide a holistic view of the metabolic processes occurring in plants under different Fe status.

## Results

### IEF-PAGE electrophoresis

**Figure 1:**
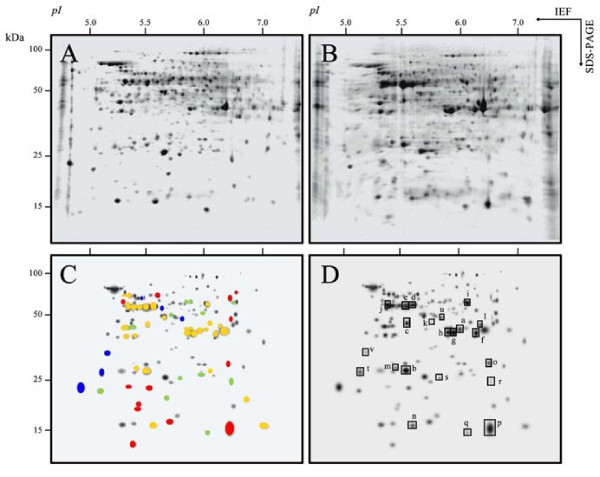
**2-D IEF-SDS PAGE proteome maps of root tips from Fe-sufficient and Fe-deficient *Beta vulgaris *plants**. Proteins were separated in the first dimension in linear (pH 5-8) IPG gel strips and in the second dimension in 12% acrylamide vertical gels. Scans of real typical gels of root tips from Fe-sufficient and Fe-deficient plants are shown in A and B, respectively. Virtual composite images, showing all polypeptides present in root tips from Fe-deficient and Fe-sufficient plants are shown in panels C and D. Statistical significance was assessed with a t-test (p < 0.10), and a 2-fold change in spot intensity between treatments was used as a threshold. Only spots that fit both criteria are marked in panel C; spots whose intensities decrease or disappear completely with Fe deficiency were labelled with green and blue marks, respectively, and those increasing with Fe deficiency or only present in Fe-deficient gels were labelled with yellow and red marks, respectively. In panel D, polypeptides that had significant homologies with proteins in the databases (using MALDI-MS and MASCOT) are marked by squares and labelled with lower-case letters. Homologies are described in detail in Table 1.

The polypeptide pattern of root tip extracts was obtained by 2-D IEF-SDS PAGE electrophoresis. Real scans of typical 2-D gels are shown in Figure 1; an average number of 141 and 148 polypeptides were detected in Fe-sufficient and Fe-deficient plants, respectively (Figure 1A and 1B). The total number of spots detected was relatively low when compared to other proteomic studies [16,17]. Several causes may account for this discrepancy, including i) protein extraction method and amount of protein loaded in the gels, ii) gel size, iii) pI range and iv) sensitivity of the staining method. Averaged 2-D polypeptide maps were obtained using gels of three independent preparations, each from a different batch of plants (gels obtained with different root tip extract preparations isolated from different batches of plants were very similar; data not shown). To better describe polypeptide changes we built a composite averaged virtual map containing all spots present in both Fe-deficient and control root tip extracts (Figure 1C and 1D). Iron deficiency caused 2-fold increases in 29 spots (yellow marks in Figure 1C, p < 0.10 student t-test) and 2-fold decreases in signal intensity in 13 spots (green marks in Figure 1C, p < 0.10 student t-test).

Furthermore, 6 spots were only detected in Fe-sufficient plant samples (blue marks in Figure 1C) and 13 spots were only detected in Fe-deficient plants (red marks in Figure 1C). All polypeptides in the composite averaged map are depicted again in Figure 1D, to permit annotation of those polypeptides where identification was achieved by matrix assisted laser desorption ionization - time of flight MS (MALDI TOF; marked by squares in Figure 1D). These polypeptides were labeled from *a *to *v *as described in Figure 1D, and homologies found are described in detail in Table 1.

**Table 1:** Proteins identified by MALDI-MS in 2-D IEF-SDS PAGE gels

Spot	Th. MW	Th. pI	Exp. MW	Exp. pI	Score^(1,2)^	Accession #	Homology	Species
Increased proteins in Fe-deficiency								

*a*	39	7.6	42	5.9	76^1^	T48396	fructose 1,6-bisphosphate aldolase	*A. Thaliana*
*b*	27	5.5	35	5.4	143^1^	gi|556171	triose-phosphate isomerase	*C. japonica*
*c*	31	4.9	45	5.4	103^1^	gi|28172909	cytosolic 3-phosphoglycerate kinase	*T. Aestivum*
*d*	49	5.6	59	5.5	188^1^	gi|1087071	enolase	*M. crystallinum*
*e*	49	5.6	58	5.4	123^1^	T12341	enolase	*L. sativa*
*f*	36	5.9	40	6.2	140^1^	CAB61618	malate dehydrogenase	*B. vulgaris*
*g*	36	5.9	40	5.8	77^1^	CAB61618	malate dehydrogenase	*B. vulgaris*
*h*	22	7.6	40	6.0	124^1^	gi|48375044	malate dehydrogenase	*N. tabacum*
*i*	55	6.0	60	6.0	185^1^	O78692	F1 ATPase α subunit	*B. vulgaris*
*j*	49	5.1	58	5.3	170^1^	gi|4388533	F1 ATPase β subunit	*S. bicolor*
*k*	36	5.2	49	5.5	299^1^	gi|1052973	fructokinase	*B. vulgaris*
*l*	41	6.5	44	6.3	102^1^	gi|38636526	formate dehydrogenase	*Q. robur*
*m*	26	5.5	35	5.4	113^1^	gi|21689609	At1g79210/YUP8H12R_1	*A. thaliana*
*n*	17	5.9	17	5.5	116^2^	gi|16301	glycine rich protein	*A. thaliana*

New spots in Fe-deficiency								

*o*	37	7.1	36	6.6	101^1^	gi|19566	glyceraldehyde 3-phosphate DH	*M. quinquepeta*
*p*	23	8.7	16	6.6	65^2^	Q9XH32	DMRL synthase	*S. oleracea*

Decreased spots in Fe-deficiency								

*q*	16	6.3	15	6.4	166^1^	gi|3309053	nucleoside diphosphate kinase I	*M. crystallinum*
*r*	23	6.4	30	6.8	217^1^	gi|11496133	oxalate oxidase-like germin 171	*B. vulgaris*
*s*	22	6.1	32	5.7	188^1^	gi|34365651	At4g27270	*A. thaliana*

Missing spots in Fe-deficiency								

*t*	23	6.4	34	5.7	217^1^	gi|11496133	oxalate oxidase-like germin 171	*B. vulgaris*
*u*	9	6.0	49	5.7	49^2^	gi|2956703	peroxidase	*S. oleracea*
*v*	29	5.1	38	5.3	69^2^	gi|5101868	caffeoyl CoA O-methyltransferase	*Z. Mays*

Scores (equal to -10*Log P, P being the probability that the observed match is a random event) are based on: ^(1) ^MS1 Peptide mass fingerprint data, with protein scores >76 being statistically significant (p < 0.05), and ^(2)^MS^2 ^ion sequencing data, with individual ion scores > 40 indicating identity or extensive homology (p < 0.05). In both cases, protein scores are derived from ion scores as a non-probabilistic basis for ranking protein hits. Proteins are separated in different groups depending on whether they increased, decreased, appeared (new) or disappeared (missing) when compared to control plants.

From the 29 spots that showed increases in signal in root tip extracts of Fe-deficient as compared to Fe-sufficient controls, the 20 more abundant were excised and analyzed by MALDI-MS. Since the sugar beet genome has not been sequenced yet and few sequences are available in the databases, identification was performed by homology searches with proteins from other plant species. From the 20 spots analyzed, 14 proteins were identified (proteins labeled *a *to *n *in Figure 1D and Table 1). These include proteins related to glycolysis such as fructose 1,6-bisphosphate aldolase (spot *a*), triose-phosphate isomerase (spot *b*), 3-phosphoglycerate kinase (spot *c*) and enolase (spots *d *and *e*, respectively). Three spots gave significant matches to malate dehydrogenase (MDH; spots *f-h*), and two more polypeptides presented homology with α and β subunits from F1 ATP synthase (spots *i *and *j*). Other proteins increasing in root tip extracts from Fe-deficient sugar beet plants as compared to the controls were fructokinase (spot *k*) and formate dehydrogenase (spot *l*). Also, one spot gave significant matches to a cytosolic peptidase At1g79210/YUP8H12R_1 (spot *m*). Spot *n *gave significant match to a glycine rich protein, which possibly has a role in RNA transcription or processing during stress conditions.

From the 13 spots detected *de novo *in proteome maps from root tip extracts of Fe-deficient plants (Figure 1C), the 6 more abundant were excised and analyzed by MALDI-MS, resulting in only 2 positive matches (spots *o *and *p *in Figure 1D and Additional file 1). These significant matches were found for glyceraldehyde 3-phosphate dehydrogenase (GADPH; spot *o*) and DMRL (spot *p*). Changes in the amount of DMRL as well as *DMRL *gene expression and flavin analysis were further studied using root tip extracts of Fe-sufficient, Fe-deficient and Fe-resupplied sugar beet plants (see below).

From the 13 spots showing a decrease in signal intensity in root tip extracts from Fe-deficient plants as compared to controls (Figure 1C), 3 were identified by MALDI-MS. Spots *q *and *r *(Figure 1D, Table 1) gave a significant match to nucleoside diphosphate kinase I and to oxalate oxidase-like germin, respectively. Spot *s *presented homology with the At4g27270 protein (Figure 1D) whose molecular function is to interact selectively with FMN, and also presents oxidoreductase activity.

From the 6 spots not detected in root tip extracts from Fe-deficient plants as compared to the controls (Figure 1C), 3 were identified by MALDI-MS (spots *t-v *in Figure 1D and Additional file 1). Proteins matched were oxalate oxidase (spot *t*), peroxidase (spot *u*) and caffeoyl CoA O-metyltransferase (spot *v*).

### DMRL synthase gene expression analysis

**Figure 2:**
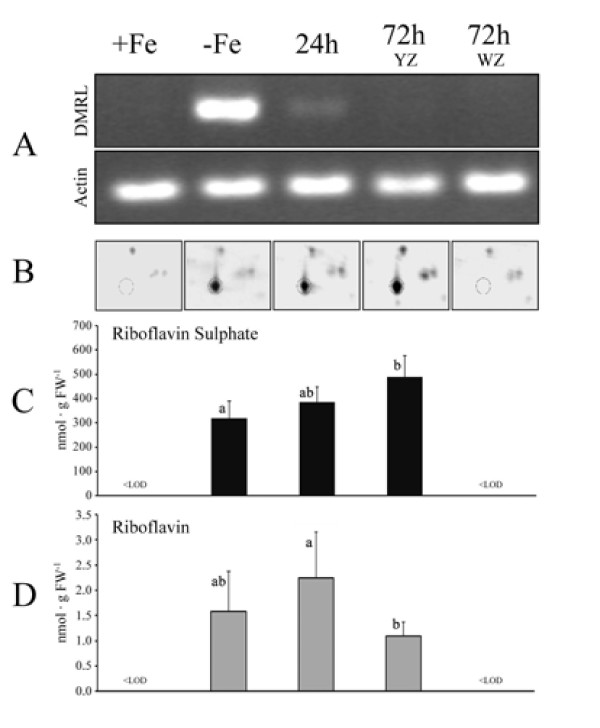
**DMRL and flavin analysis**. Semi-quantitative RT-PCR analysis of the *BvDMRL *and actin gene transcripts (A), zoom scans of typical gels containing the BvDMRL protein (B) and riboflavin sulphates (C) and riboflavin (D) concentrations in sugar beet root tips from Fe sufficient (+Fe), Fe-deficient (-Fe), 24 h Fe-resupplied, 72 h Fe-resupplied YZ and 72 h Fe-resupplied WZ. Letters in (C) and (D) indicate significant differences (n = 6, p < 0.05, Duncan test).

To identify a putative DMRL synthase cDNA in *B. vulgaris*, primers were designed based on DMRL synthase sequence from the closely related specie *S. oleracea *(AF147203.1). A PCR product of approximately 675 bp was amplified, purified and sequenced (Additional file 2A). The translated amino acid sequence for the DMRL synthase putative ORF had 224 aminoacids, a predicted MW of 23.7 kDa and a pI of 8.26. The predicted protein displayed 90% identity at the amino acid level with *S. oleracea *DMRL (AAD44808.1), and homologies with other known DMRLs sequences included in the alignment analysis ranged from 50 to 56% (Additional file 2B). Thus, we have assigned the name *BvDMRL *to the corresponding DMRL synthase gene of *B. vulgaris*, and the sequence has been deposited in GenBank as accession GQ375163. Transcriptional regulation by Fe status of the *BvDMRL *gene was assessed by semi-quantitative RT-PCR. *BvDMRL *transcript abundance was much higher in root tips from Fe-deficient as compared to control plants (Figure 2A). The expression of *BvDMRL *was drastically reduced 24 h after Fe-resupply to Fe-deficient plants (Figure 2A) but it was still higher than that observed in Fe-sufficient root tips. However, transcript abundance in root tips sampled 72 h after Fe-resupply, which showed two different zones (white -WZ- and yellow -YZ-, see [26]), had a low expression level, similar to that measured in Fe-sufficient plants (Figure 2A).

### DMRL synthase protein analysis

Real scans of the 2-D gel zone where DMRL synthase protein is located (16 kDa, pI 6.6) are shown in Figure 2B. The DMRL synthase protein was detected not only in Fe-deficient root tip extracts but also in extracts of root tip YZ from plants resupplied with Fe for 24 and 72 h (Figure 2B). Conversely, DMRL synthase was not detected in extracts of the new WZ of root tips from plants 72 h after Fe resupply, or in Fe-sufficient root tip extracts (Figure 2B).

### Flavin concentrations

Since DMRL synthase is one of the key enzymes in flavin synthesis, we also examined flavin concentrations in root tip extracts from Fe-sufficient, Fe-deficient and Fe-resupplied plants by high performance liquid chromatography (HPLC). Riboflavin, FAD and Rbfl sulphates were found in Fe-deficient and Fe-resupplied root tip YZ extracts. Riboflavin sulphates and Rbfl account for 98% and 2% of the total flavin concentration, respectively, and only traces of FAD were found. Rbfl sulphate concentrations were 319, 384 and 488 nmol g^-1 ^FW in the root YZ of Fe-deficient plants and 24 h and 72 h Fe-resupplied plants, respectively (Figure 2C). Riboflavin concentration ranged from 1.1 to 2.3 nmol g^-1 ^FW in root YZ (Figure 2D). Only traces of these compounds were present either in controls or in the WZ of 72 h Fe-resupplied plants.

### Metabolite Analyses

**Figure 3:**
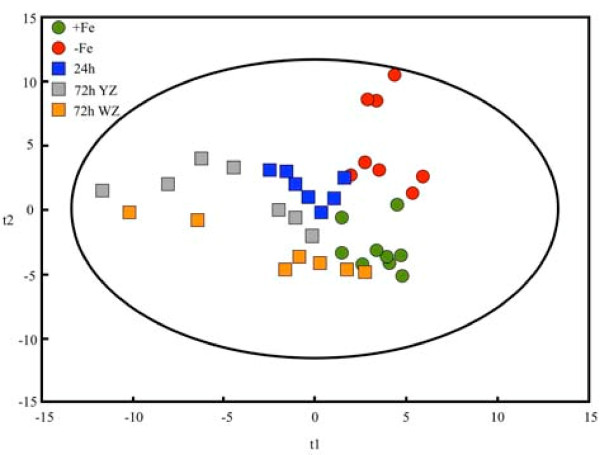
**Score scatter plot of identified metabolites**. Partial Least Square (PLS) analysis, showing the score scatter plot of PLS vector 1 (t1) vs. PLS vector 2 (t2) of identified metabolites in sugar beet root tips from Fe sufficient (+Fe, green circles), Fe-deficient (-Fe, red circles), 24 h Fe-resupplied (24h, blue squares), 72 h Fe-resupplied YZ (72h YZ, grey squares) and 72 h Fe-resupplied WZ (72h WZ, orange squares).

Changes induced by Fe-deficiency and Fe-resupply in the root tip metabolome were evaluated by non-biased gas chromatography mass spectrometry (GC-MS) metabolite profiling. A total of 326 metabolites were present in at least 80% of the samples of at least one treatment, and 77 of them were identified. Partial least square analysis shows a good separation between +Fe and -Fe root tips (Figure 3). Iron-deficient samples were closer to the 24 h and 72 h YZ samples than to the 72 h WZ ones. On the other hand, the 72 h WZ samples were closer to the +Fe samples than to the -Fe ones.

Iron deficiency and/or resupply caused significant changes (p < 0.05) in the levels of 62 out of the 77 identified metabolites. Metabolite level data were normalized to the mean response of the +Fe treatment; response ratios, defined as the level in a given treatment divided by the level in the +Fe control, are indicated in Table 2. Iron deficiency caused significant changes in the response ratios of 26 metabolites. Large (> 4-fold) increases were found for some organic acids (citric and aconitic acid), some sugars (sucrose, myo-inositol and those of the raffinose family of oligosaccharides -RFOs-, namely galactinol and raffinose), nicotianamine and 2-aminoadipic acid. The response ratio of oxalic acid decreased markedly in -Fe conditions, whereas those of other aminoacids, N compounds, lipid metabolites and others did not show large (> 4-fold) changes when compared to the Fe-sufficient controls.

**Table 2:** Identified metabolite response ratios of the different treatments *vs*. Fe-sufficient controls (+Fe)

#	metabolites	-Fe	24h	72hYZ	72hWZ	*A. thaliana *KEGG pathway number
Aminoacid and Nitrogen Metabolism						

1	nicotianamine	**7.9**	**11.8**	**2.9**	**2.1**	
2	2-aminoadipic acid	**6.4**	**3.5**	**12.3**	11.2	0300, 1100, 0310
3	2-hydroxyglutaric acid	**3.8**	**4.1**	**2.3**	1.3	0650
4	citrulline	**3.6**	1.4	**2.9**	1.7	0330, 1100
5	lysine	**3.0**	**2.2**	2.5	**-1.2**	0300, 1100, 0310, 0780, 0960, 0310
6	serine	**2.4**	**1.7**	**1.8**	**-1.8**	0970, 1100, 0260, 0600, 0460, 0271, 0272, 0920, 0680
7	hydroxylamine	1.1	**2.0**	**4.2**	**3.2**	0910
8	urea	1.0	1.0	**2.5**	6.2	0330, 1100, 0230, 0240
9	arginine + ornithine	2.2	1.1	1.6	0.0	0330, 1100, 0970
10	aspartic acid	1.0	1.3	1.4	1.1	0970, 1100, 0260, 0760, 0710, 0300, 0910, 0252, 0460, 0410, 0330, 0770
11	glutamic acid	1.1	1.0	1.2	-1.0	0970, 1100, 0480, 0860, 0650, 0251, 0340, 0330
12	glycine	-1.3	-1.3	1.3	-1.5	0970, 1100, 0260, 0480, 0730, 0310, 0230, 0120, 0460, 0860, 0680
13	phenylalanine	1.2	-1.1	1.2	-1.1	0970, 1100, 0400, 0966, 0960, 0360, 0940
14	tyrosine	2.1	-1.2	1.3	-1.3	0970, 1100, 0950, 0730, 0960, 0350, 0940, 0966
15	putrescine	-1.1	-1.8	-1.3	-1.3	1100, 0960, 0480
16	ornithine	1.6	-1.2	1.2	-1.0	1100, 0330, 0480
17	tryptophan	1.0	**-1.9**	-1.2	**-2.4**	1100, 0970, 0380, 0901, 0400, 0966
18	oxoproline	-1.3	**-1.9**	-1.1	-1.2	0330
19	valine	1.2	1.0	1.4	**-2.2**	0970, 0901, 0400, 0966, 0380, 1100
20	asparagine	-1.0	-1.8	-1.2	**-2.4**	1100, 0970, 0910, 0252, 0460
21	alanine	-1.4	1.7	1.5	**-3.4**	1100, 0970, 0252, 0710, 0450, 0430, 0272, 0720
22	glutamine	-1.6	**-4.5**	**-2.5**	**-3.8**	1100, 0970, 0910, 0251, 0240, 0230

Carbohydrate metabolism						

23	galactinol	**33.7**	**86.2**	**23.2**	7.6	0052
24	raffinose	**16.3**	**59.7**	**9.7**	9.9	0052
25	sucrose	**4.7**	**2.3**	**1.9**	2.2	0052, 1100, 0500
26	lactobionic acid	**3.3**	**10.9**	**4.8**	**3.2**	
27	N-acetyl-D-mannosamine	**2.1**	**1.5**	**2.0**	**1.6**	1100, 0530
28	arabinose	**2.1**	1.4	**2.2**	1.4	
29	xylonic acid	**2.0**	**1.6**	**2.2**	1.4	
30	inulobiose	**2.0**	**3.4**	**2.3**	2.1	
31	cellobiose	5.1	**8.0**	**4.7**	**2.2**	0500
32	mannitol	-1.3	1.6	**3.1**	1.4	0052, 1100, 0053, 4070, 0562
33	xylitol	1.1	1.3	**2.1**	1.4	1100, 0040
34	fructose	-1.8	-1.7	1.9	-1.3	0052, 1100, 0051, 0500
35	suberyl glycine	1.1	-2.0	-1.1	**-2.6**	

Co-enzymes and alkaloid biosynthesis						

36	ribitol	5.6	**5.4**	**2.9**	2.0	1100, 0040, 0740
37	pantothenic acid	1.7	**2.0**	**2.2**	1.4	1100, 0410, 0770
38	nicotinic acid	-1.3	1.3	**2.6**	1.8	1100, 0760, 0960

Glycolysis						

39	glucose-1-phosphate	1.4	**2.7**	2.5	1.7	0052, 1100, 0010, 0500, 0040, 0520
40	glucose	1.1	-3.9	1.8	-2.4	0052, 1100, 0010, 0500, 0030, 0901
41	fructose-6-phosphate	-1.1	-1.3	1.2	1.2	0710, 1100, 0040, 0680, 0530
42	glucose-6-phosphate 2	-1.3	-1.6	1.2	-1.1	0500, 1100, 0562
43	3-phosphoglycerate	**-1.9**	-1.3	**1.5**	1.2	0561, 0260, 0010, 0710, 0630, 1100

Glyoxylate and dicarboxylate metabolism						

44	glyceric acid	**3.4**	1.3	**1.5**	1.7	0561, 0260, 0030, 0630, 1100
45	glycolic acid	2.9	2.0	3.1	**3.6**	0630, 1100, 0361
46	oxalic acid	**-10.9**	**-5.2**	**-3.6**	1.5	0630, 1100

Lipid metabolism						

47	behenic acid	**1.9**	**1.9**	**2.8**	**3.3**	1040
48	y hexaric acid	**1.6**	**3.8**	**3.2**	1.7	
49	stearic acid	-1.2	**2.8**	4.1	**3.3**	0061, 1040
50	glycerol	-1.2	**2.2**	**3.9**	**3.8**	
51	cerotic acid	-1.1	**2.0**	**3.8**	**2.9**	
52	myristic acid	1.0	**2.1**	**3.7**	**2.7**	0061
53	palmitic acid	-1.2	**1.9**	**3.4**	**2.7**	0061, 1040, 1100, 0062, 0071
54	phosphoethanolamine	1.7	**1.7**	1.5	1.7	0600, 0260, 1100, 0564
55	lauric acid	-1.1	2.1	**3.5**	2.3	0061
56	capric acid	-1.1	1.4	**3.1**	**2.1**	0061
57	pentadecanoic acid	1.0	1.2	2.9	3.0	
58	pelargonic acid	-1.2	1.1	2.5	1.8	
59	linoleic acid	-1.4	1.2	2.3	2.4	1040, 1100
60	glycerol-alpha-phosphate	-1.0	1.0	1.5	1.5	

Oxidative stress						

61	threonic acid	**3.7**	1.6	1.2	1.7	
62	dehydroascorbate	**3.3**	1.4	**2.8**	1.4	
63	2-hydroxypentanoic acid	-1.2	1.5	**3.5**	**1.9**	0982
64	1,2,4-benzenetriol	1.5	3.0	**4.5**	**4.6**	1100, 0361, 0362, 0627
65	y pentonic acid	1.6	-1.4	-1.1	-1.8	

Pentose phosphate pathway						

66	ribose	1.5	**1.7**	**1.6**	1.9	1100, 0030
67	gluconic acid	1.6	**1.7**	2.5	1.6	1100, 0030, 0710, 0230

Signaling						

68	myoinositol	**5.3**	**2.2**	**3.3**	2.4	
69	GABA	1.8	**4.4**	4.5	1.5	1100, 0251, 0410, 0650
70	inositol-monophosphate	-1.2	**1.7**	**2.3**	**2.2**	4070, 0562

TCA Cycle						

71	aconitic acid	**4.5**	**5.4**	**5.3**	1.8	1100, 0020, 0720, 0630
72	citric acid	**21.1**	**16.2**	**10.3**	1.5	1100, 0020, 0720, 0630, 0251, 0252
73	malate	**3.3**	**2.6**	**2.6**	1.8	1100, 0020, 0720, 0630, 0251, 0252, 0710, 0620
74	succinic acid	1.4	1.5	**3.7**	2.2	1100, 0020, 0720, 0630, 0251, 0252, 0350, 0650, 0632, 0361, 0190, 0640

Others						

75	acetohydroxamic acid	1.1	-1.6	**5.0**	**3.7 **	
76	phosphoric acid	-1.3	**-4.6**	-1.5	3.8	1100, 0190, 0195, 0550
77	adenosine-5-monophosphate	**-1.3**	-1.2	1.3	1.1	1100, 0230, 0908

When the response ratio (level in a given treatment divided by the level in the +Fe treatment) was lower than 1 the inverse was taken and the sign changed. Values indicated in bold represent a t-test significance of p < 0.05. The last column shows *A. thaliana *KEGG pathway numbers where the metabolite is predicted to be involved; all KEGG pathway numbers are hyper-linked to the corresponding KEGG website. To reach one specific pathway in KEGG type in your web browser:  "http://www.genome.jp/kegg-bin/show_pathway?ath0XXXX" being XXXX the pathway number.

Twenty-four hours after Fe-resupply, there was a dramatic coordinated increase in the root tip response ratios of galactinol, raffinose, lactobionic acid, cellobiose and nicotianamine when compared to those found in Fe-deficient roots, whereas the response ratios of sucrose, myo-inositol, citrate and malate decreased. Seventy-two hours after Fe resupply, the response ratios of galactinol, raffinose, cellobiose, nicotianamine and many other compounds had decreased in the YZ areas, whereas in the WZ the response ratios were very low. The response ratio of many of the lipids increased moderately in all Fe resupplied samples. Metabolites in the coenzyme, glycolysis, oxidative stress, pentose phosphate pathway and signalling categories did not show large response ratio changes with Fe resupply.

Regarding the unknown metabolites, significant changes (at p < 0.01, with response ratios > 4) were found for 46 of the 269 metabolites with Fe deficiency and/or resupply (Additional file 1). BinBase metabolite ID 211891 showed the largest response ratio (18, 146 and 70-fold, for -Fe, 24 h and 72 h YZ, respectively). The response of this metabolite to Fe status was very similar to that found for the RFOs, and in fact its mass spectra and retention index show a good similarity in BinBase to those of other disaccharides such as leucrose, suggesting that it could be a RFO related compound (to see 211891 mass spectra, go to http://eros.fiehnlab.ucdavis.edu:8080/binbase-compound/, select the rtx5 database and type 211891).

### Raffinose and galactinol concentrations

The concentrations of raffinose and galactinol in root tips were determined by high performance liquid chromatography HPLC-MS. Raffinose concentrations were 35.1, 50.4, 35.4, 80.8 and 4.2 nmol g FW^-1 ^in the -Fe, 24h, 72h WZ, 72h YZ and +Fe tissues, respectively. Galactinol concentrations were 75.6, 39.8, 16.1, 37.1 and 7.6 nmol g FW^-1 ^in the -Fe, 24h, 72h WZ, 72h YZ and +Fe tissues, respectively.

## Discussion

The changes induced by Fe deficiency in the root tip proteome and metabolome from sugar beet plants grown in hydroponics have been studied. More than 140 proteins (Figure 1) and 300 metabolites (Table 2 and Additional file 1) were resolved in sugar beet root tip extracts. Iron deficiency resulted in significant and higher than 2-fold changes in the relative amounts of 61 polypeptides, and 22 of them were identified. Out of 77 identified metabolites, 26 changed significantly with Fe deficiency. In general, our results are in agreement with previous transcriptomic [14], proteomic [15-17] and enzymatic [19] studies on Fe-deficient roots. Our data confirm the increases previously found in proteins and metabolites related to carbohydrate metabolism and TCA cycle pathways [9,13,27]. Two major changes induced by Fe deficiency in roots are described in this study for the first time: the increase in DMRL synthase protein concentration and gene expression, and the increase in RFO sugars.

The largest change found in the proteome map of root tip extracts from sugar beet plants grown in Fe deficiency conditions corresponded to DMRL synthase, which was detected *de novo *in Fe-deficient root tips, and is the protein with the highest concentration in these gels (spot *p *in Figure 1D). This enzyme catalyses the fourth step of Rbfl biosynthesis, and Rbfl is the precursor of Rbfl sulphates, FMN and FAD, the last one being a cofactor for the root plasma membrane Fe reductase [10]. The expression of *BvDMRL *decreased drastically 24 h after the addition of Fe to Fe-deficient plants, whereas DMRL synthase protein abundance and Rbfl and Rbfl sulphate concentrations did not change significantly with Fe-resupply in the YZ of root tips (Figure 2), suggesting that the turnover of this protein is slow. Accumulation in Fe-deficient roots of flavin compounds, including Rbfl and Rbfl 3' - and 5'-sulphate is a characteristic response of sugar beet and other plant species [6]. The exact role of flavins in Fe deficiency is unknown, and it has been hypothesized, based on the similar location of flavin accumulation and Fe reduction and on the fact that the Fe reductase is a flavin-containing protein, that free flavin accumulation may be an integral part of the Fe-reducing system in roots from Strategy I plants [9,28]. On the other hand, these compounds are secreted to the growth media at low pH [6] and, assuming high concentrations at the secretion site, they could mediate extracellular electron transfer between soil Fe deposits and root Fe reductase as it has been described for flavin phosphates secreted by some bacteria [29]. Moreover, excreted flavins could also act as a plant-generated signal that could influence rhizosphere microbial populations, indirectly affecting Fe availability [11].

A major change in carbohydrate metabolism was the large increase in RFO compounds (34- to 16-fold changes in the response ratios of galactinol and raffinose, respectively; Table 2) that occurs in roots with Fe deficiency. This increase was higher than that found for sucrose (5-fold). The total concentrations of raffinose and galactinol were also determined by HPLC-MS, and concentrations of both compounds in the 35-80 nmol g FW^-1 ^range were found in Fe-deficient and Fe-resupplied root tips (equivalent to approximately 50 μM), whereas concentrations in the +Fe treatment were one order of magnitude lower. The sum of the raffinose and galactinol concentrations in the -Fe, 24h, 72hWZ, 72hYZ and +Fe tissues was 13.9, 7.4, 2.2, 5.1 and 0.6% of the total sucrose, respectively, supporting the relevance of the RFOs changes with Fe status. RFOs have diverse roles in plants, including transport and storage of C [30] and acting as compatible solutes for protection in abiotic stresses [31,32]. Other explanation for the large increase in the relative amounts of RFOs could be the ability to function as antioxidants [33,34]; galactinol and raffinose have hydroxyl radical scavenging activities similar to other soluble antioxidants such as glutathione and ascorbic acid [33]. Since ROS damage and ROS detoxification strategies have been observed in Fe-deficient roots [25,35], the increase in RFO concentration could help to alleviate ROS damage produced under Fe deficiency. Moderate increases in sugars commonly found in cell walls such as cellobiose, xylonic acid and arabinose, which may indicate cell wall modifications, were measured in sugar beet Fe-deficient root tips. Changes in cell wall metabolism have been also described in Fe-deficient tomato roots [16]. On the other hand, it has been described that cell wall damage generates oligosaccharides that can act as signalling molecules in stresses such as wounding [36]. The increase in RFOs could also act as a long distance Fe-deficiency signal via phloem sap transport. This is the first description of RFOs accumulation in plants under Fe deficiency, and the physiological implications of this increase deserve further consideration.

Most of the proteins found to be up-accumulated in sugar beet root tips by Fe deficiency were identified as carbohydrate catabolism enzymes, including 5 of the 10 glycolytic pathway enzymes (fructose 1,6-bisphosphate aldolase, triosephosphate isomerase, glyceraldehyde 3-phosphate dehydrogenase, 3-phosphoglycerate kinase and enolase), one of the citric acid cycle (MDH) and fructokinase. Increases in the activities and concentrations of several glycolytic enzymes in root extracts with Fe deficiency have been previously found, including fructose 1,6-bisphosphate aldolase, enolase, triosephosphate isomerase and GADPH [15,16,37-39]. Also, increases in the activities and concentrations of several enzymes of the citric acid cycle with Fe deficiency have been previously reported in root extracts, including MDH [9,16,38]. Results are also in agreement with microarray gene analysis in Fe-deficient *A. thaliana *roots [14]. Increases in the amount of PEPC have been found at the protein level [16,40,41], but this enzyme, with a molecular mass of 110 kDa, was not in the range (apparent molecular mass 10-100 kDa) used in our 2-D gels. Up-regulation of carbohydrate catabolism in roots of plants grown in Fe deficient conditions is probably a result of an increased demand of energy and reducing power in roots needed to sustain the increased activity of H^+^-ATPase and Fe reductase [14,16,19]. Also, two spots corresponding to different subunits of F1 ATP synthase increased in 2-D gels from Fe deficient root tips, further supporting the higher energy requirement in these roots. Moreover, our results show an increase in the amount of formate dehydrogenase, an enzyme related to the anaerobic respiration, in Fe-deficient roots, confirming the results of enzyme [9] and transcriptional [14] analysis. Anaerobic respiration is an alternative pathway for energy production when oxidative phosphorylation is impaired.

Metabolite studies revealed large increases in organic acids, including a 20-fold citric acid increase. These increases in TCA cycle organic acids with Fe deficiency are coupled with increases in glycolysis [9,16] and root C fixation by PEPC [40,41], and provide an anaplerotic, non-autotrophic *(via *xylem) C source for leaves which have otherwise reduced photosynthetic rates [18,22]. Malate and citrate could also be pumped from the cytosol to the mitochondria via a di-tricarboxylate carrier (DTC) where they would allow a higher turnover of reducing equivalents [23]. A significant decrease (-10.9) in oxalic acid concentration was observed in Fe deficient root tips, and similar decreases (*ca.* 10 times) have been reported in Fe-deficient tomato roots [13]. The implications of oxalate concentration decreases with Fe deficiency are still not known, since the role of oxalic acid in plants is quite different from that of the other organic acids, and for a long time it has been considered as a toxin or a metabolic end product [42]. Regarding N and amino-acid compounds, a large increase (8-fold) was measured for nicotianamine, which has been described to play a role in cytosolic Fe availability [43,44].

**Figure 4:**
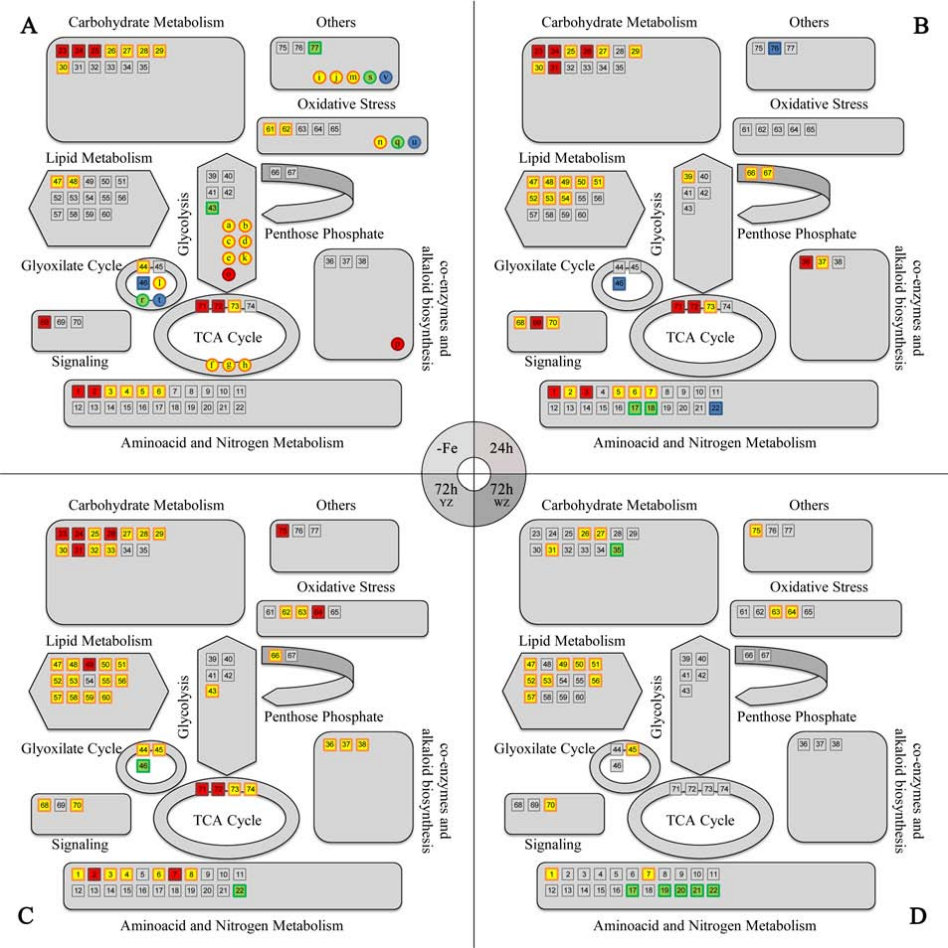
**Changes in metabolic pathways as affected by Fe status**. Pathways related to metabolites and proteins were integrated according to the KEGG database. Statistical t-student tests were performed to both protein and metabolite data to show statistically relevant changes between samples. Red squares indicate metabolites showing significant, marked (more than 4-fold) up-accumulation compared to the controls, and yellow ones mean significant, moderate (less than 4-fold) up-accumulation. Blue squares mean significant, marked (more than 4-fold) down-accumulation, and green ones mean significant moderate (less than 4-fold) down-accumulation. The same colour code was used with proteins, represented by circles. Red circles are proteins detected only in Fe-deficient gels, and yellow ones mean significantly up-accumulated proteins using a 2-fold threshold. Decreased (at least 50%) and missing proteins in the Fe-deficient treatment were represented in green and blue respectively. Each number or letter corresponds to those shown in the corresponding protein and metabolite tables (Tables 1 and 2 respectively). Panels correspond to root tip extracts from plants Fe-deficient (A), 24h after Fe resupply (B), and 72h after Fe resupply (YZ in panel C and WZ in panel D).

A comprehensive representation of the metabolomic and proteomic changes taking place in root tips under Fe deficiency and resupply is shown in Figure 4. Red and yellow symbols indicate major and moderate increases in metabolites (squares) and proteins (circles) compared to the Fe-sufficient controls. Blue and green symbols indicate major and moderate decreases in metabolites (squares) and proteins (circles) compared to the controls. Besides the major increases in RFOs and DMRL, Fe deficiency induced significant changes in root tip metabolism, mainly associated to increases in carbohydrate catabolism, glycolysis and TCA cycle and to a lesser extent in aminoacid and nitrogen metabolism (Figure 4). Similar changes were observed in the 24 and 72h YZ Fe-resupplied roots, whereas the WZ of 72 h Fe-resupplied plants did not show major changes when compared to +Fe plants (Figure 4). On the other hand, the relative amount of lipid metabolism compounds did not change markedly in Fe-deficient roots, whereas Fe resupply caused a moderate increase in this type of metabolites (Figure 4).

## Conclusions

The proteomic and metabolic profiles of root tips from sugar beet plants were affected by Fe deficiency and resupply. Changes in the glycolysis and TCA cycle metabolites and proteins confirmed previous studies. Two novel and major findings were the increase in i) DMRL synthase protein concentration and gene expression, and ii) some RFO sugars (raffinose and galactinol). These new findings give new perspectives to the knowledge of Fe deficiency.

## Methods

### Plant Material

Sugar beet (*Beta vulgaris *L. "Monohil" and "Orbis" from Hilleshög, Landskröna, Sweden and Syngenta, Madrid, Spain, respectively) was grown as described elsewhere [26]. "Monohil" was always used, with the exception of raffinose and galactinol analysis, which was carried out with "Orbis". After seed germination in vermiculite and 2 weeks in half-strength Hoagland's nutrient solution with 45 μM Fe(III)-EDTA, plants were transferred into 20 L plastic buckets (four plants per bucket) containing half-strength Hoagland's nutrient solution with either 0 or 45 μM Fe(III)-EDTA. The pH of the Fe-free nutrient solution was buffered at approximately 7.7 by adding 1 mM NaOH and 1 g L^-1 ^of CaCO_3_. In the Fe resupply experiments, plants grown for 10 d in the absence of Fe were transferred to 20 L plastic buckets containing half-strength Hoagland's nutrient solution, pH 5.5, with 45 μM Fe(III)-EDTA. The root subapical region from Fe-sufficient plants (+Fe), Fe-deficient plants (-Fe), Fe-deficient plants resupplied with Fe for 24 h (24h) and Fe-deficient plants resupplied with Fe for 72 h (72h) was collected with a razor blade and immediately frozen in liquid N_2_. The specific regions of root sampled were: in the case of +Fe, -Fe and 24 h plants, the first 10 mm from the root apex (+Fe roots were thin and white, whereas -Fe and 24 h roots were swollen and yellow); in the case of 72 h Fe resupplied roots two zones were sampled separately, the first 5 mm from the root apex, where a new white zone had developed (72h WZ), and the next 5 mm, comprising the still swollen and yellow root zone (72h WZ). Samples were taken at approximately 4 h after light onset in the growth chamber.

### Protein extraction and analysis

Protein extracts were obtained as described elsewhere [45] and protein concentration was measured with RC DC Protein Assay (Bio-Rad, Hercules, CA, USA). A first dimension isoelectric focusing (IEF) separation was carried out on ReadyStrip IPG Strips (BioRad), using a linear pI gradient 5-8. Strips were loaded in a PROTEAN IEF Cell (BioRad) and focused at 20°C, for a total of 14000 V.h. For the second dimension polyacrylamide gel electrophoresis (SDS-PAGE), IPG strips were placed onto 12% SDS-PAGE gels to separate proteins between 10 and 100 kDa. Proteins were stained with Coomassie-Blue R-250 (Sigma, Barcelona, Spain) and results analyzed with the PDQuest 8.0 software (BioRad) [46]. Gels were made from independent root tip preparations from three different batches of plants. In-gel digestion, sample preparation, MALDI TOF and MALDI TOF-TOF peptide mass fingerprint and database searching was carried out as described in detail elsewhere [46]. Statistical significance was tested with a t-Student test (p < 0.10). A 2-fold change in spot signal intensity between treatments was taken as a threshold.

### Metabolite extraction, analysis, data processing and statistical analysis

Metabolite extraction and analysis was carried out as described previously [47]. Root tips (*ca. *50 mg FW) from eight different replicates per treatment (one plant each) were used. After metabolite extraction and derivatization, samples (1 μL) were injected randomly in split-less mode with a cold injection system (Gerstel, Mülheim an der Ruhr, Germany) and analyzed by GC (Agilent 6890, San Jose, CA, USA) using a Rtx 5Sil MS column (30 m x 0.25 mm, 0.25 μm film thickness) and an integrated guard column (Restek, Bellefonte, PA, USA). The GC was connected to a Leco Pegasus IV TOFMS spectrometer controlled with Leco ChromaTOF software v.2.32 (Leco, St. Joseph, MI, USA). Initial peak detection and mass spectra deconvolution were performed with Leco Chroma-TOF software v.2.25. GC-MS chromatograms were processed as described previously [47]. Further analysis after deconvolution was done using the semi-automated workflow in the UC Davis Genome Center metabolomics laboratory [48]. Metabolite data were normalized using FW and the sum of all metabolite heights in a single run to account for small FW and GC injection variations [47]. Statistical analysis, including i) breakdown one-way ANOVA univariate statistics (p < 0.05), ii) multivariate analysis supervised partial least square (PLS) and iii) unsupervised principal component analysis (PCA; data not shown) were carried out with Statistica software (v.8.0. StatSoft, Inc., Tulsa, OK, USA). A 4-fold change in signal intensity between treatments was taken as a threshold for discussion.

### DMRLs gene identification and expression analysis

To identify putative dimethyl-8-ribityllumazine (DMRL) synthase gene sequences in sugar beet, searches were performed with TBLASTX [49] using previously identified DMRL synthase sequences from *A. thaliana *(AF148649.1), *Nicotiana tabacum *(AF422802.1) and *Spinacia oleracea *(AF147203.1). Since the search gave no hits in *B. vulgaris*, primers were designed based on the DMRL synthase sequence from *S. oleracea *(AF147203.1), the closest phylogenetically related species. Primers used to amplify the complete cDNA sequence were f-ATGGCTTCATTTGCAGCTTCT and r-TTAGGCCTTCAAATGATGTTC.

Total RNA from Fe-deficient root tips was isolated using the RNeasy Plant Mini kit (Qiagen, GmbH, Hilden, Germany) according to the manufacturer's instructions. The concentrations of RNAs were assessed by ultraviolet and visible absorption spectroscopy (UV/Vis). The structural integrity of the RNAs was checked with non-denaturing agarose gel and ethidium bromide staining. A sample aliquot containing 3 μg total RNA was subjected to reverse transcription with 25 μg mL^-1 ^oligo (dT) primer, 0.05 mM dNTP mix and 1 unit of Superscript II Reverse Transcriptase (Invitrogen, Carlsbad, USA) in a final volume of 20 μL. PCR reactions were carried out with 2 μL of resulting cDNA solution, using 0.5 μM of the specific primers, 75 mM Tris HCl pH 9.0, 1.5 mM MgCl_2_, 50 mM KCl, 20 mM (NH_4_)_2_SO_4_, 200 μM dNTP's and 0.5 units DNA polymerase (Biotools, Madrid, Spain). PCR cycling conditions were as follows: 95°C for 5 min, 30 cycles consisting of 95°C for 45 s, 55°C for 45 s and 70°C for 1 min, and a final period of 10 min at 70°C. The PCR product was purified with the QIAquick gel extraction kit (Quiagen) and sequenced (CNIO, Madrid, Spain). The nucleotide sequence was translated, MW and pI predicted by using the ExPASy server tools [50], and multiple-sequence alignments done with ClustalIW v.2 [51]. Expression of the *DMRL *gene in *B. vulgaris *root tips was analyzed by semi-quantitative RT-PCR. One plant per treatment was used to extract RNA, and two different batches of plants were analyzed. RNA extraction and RT-PCR reactions were carried out as described above. Amplified products from 15 μl of PCR reaction were visualized on 1% TBE agarose gel containing ethidium bromide. Bands were photographed using the Quantity One 4.5.1 Chemdoc EQ Software system (Bio-Rad). Actin (DQ866829.1) was used as housekeeping gene. Primers used to amplify the actin sequence were: f-GGCAAACAGGGAAAAGATGA and r-ACGACCAGCAAGATCCAAAC. RT-PCR reactions were carried twice for each sample set.

### Flavin analysis

Root material (*ca. *100 mg FW) was frozen in liquid N_2 _and ground in a mortar with 0.1 M ammonium acetate, pH 6.1. Extracts were centrifuged for 5 min at 14000 g and the supernatant stored at -80°C until analysis. Flavins were determined by HPLC-UV/Vis as described by Susín *et al. *[6]. Six samples per treatment from 3 independent batches of plants were used for measurements.

### Raffinose and galactinol analysis

Root tips (*ca.* 100 mg) from 3 different plants per treatment were extracted with 80% ethanol as described elsewhere [52]. After ethanol evaporation, extracts were resuspended in acetonitrile-H_2_O and analyzed by HPLC-MS as described in [53] with some modifications. Chromatographic separation was performed on an Alliance 2795 HPLC system (Waters, Mildford, MA, USA). The autosampler was kept at 4°C and the column compartment temperature was set at 25°C. Injection volume was 10 μL, and a ZIC-pHILIC, 150 × 2.1 mm, 5 μm column (Sequant, Umea, Sweden) was used with a flow rate of 200 μL min^-1^. The mobile phase was built using two eluents: A (5 mM ammonium acetate in 0.1% formic acid at pH 4) and B (acetonitrile 0.1% formic acid). All mobile phase chemicals were LC-MS grade (Riedel-de Haen, Seelze, Germany). For separation, the initial solvent composition 90% B and 10% A was taken to 10% B and 90% A with a 19 min linear gradient. Then, the mobile phase returned to initial conditions in 1 min and these conditions were kept for another 10 min, to allow column equilibration. Total run time per sample was 30 min. MS analysis was carried out with a electrospray MS apparatus (micrOTOF II, Bruker Daltoniks GmbH, Bremen, Germany) in the 50-1000 *m/z *range. The micrOTOF II was operated in negative mode at 3000 and -500V capillary and end-plate voltages, respectively. Capillary exit, skimmer 1 and hexapole RF voltages were set at -82.1, -41.4 and 80.0 V, respectively. Nebulizer gas (N_2_) pressure was kept at 2.0 bar and drying gas (N_2_) flow was set at 8 L min^-1 ^with a temperature of 180°C. Mass calibration was carried out with a 10 mM Li-formate solution using a syringe pump (Cole-Parmer Instruments, Vernon Hills, IL, USA). The system was controlled with the software packages MicrOTOF Control v.2.2 and HyStar v.3.2 (Bruker Daltonics). Data were processed with Data Analysis v.3.4 software (Bruker Daltonics). Quantification was carried out by external calibration with standards of sucrose, raffinose and galactinol (Sigma).

## Authors' contributions

SA and AFLM carried out the proteomic, genetic and flavin studies. RRA carried out the metabolomic and RFO studies. JRC analyzed proteomics data and prepared some of the figures. GW and OF prepared and analyzed metabolomics data. AAF and GZ analyzed critically the results. RRA, SA, AFLM, AAF and JA wrote the manuscript. All authors read and approved the final manuscript.

## Supplementary Material

Additional file 1 **Unknown metabolite response ratios of the different treatments *vs. *Fe-sufficient controls (+Fe)**. List of unknown metabolites with response ratios (level in a given treatment divided by the level in the +Fe treatment) higher than 4 and a t-test significance of P < 0.01 (indicated in bold). When the ratios were lower than 1 the inverse was taken and the sign changed. Click here for file

Additional file 2 **DMRL synthase sequence and alignment**. DMRL synthase sequence (A) in *Beta vulgaris *(GQ375163) and alignment (B) of DMRL synthase protein from *Beta vulgaris *(Bv) and *Spinacia oleracea *(So; AAD44808.1), *Nicotiana tabacum *(Nt; AAQ04061.1), *Arabidopsis thaliana *(At; AAD44810.1), *Cucumis sativus *(Cs; ABZ88150.1), *Solanum chacoense *(Sc; ACB32230.1) and *Oryza sativa *(Os; ACS94980.1) and *Zea mays *(Zm; ACG35456.1). More information about the DMRL sequences can be found at http://www.ncbi.nlm.nih.gov/protein/ Click here for file
