# Supplementary material for: Changes in the proteomic and metabolic profiles of Beta vulgaris root tips in response to iron deficiency and resupply
Source: BMC Plant Biol. 2010 Jun 21;10:120. doi: 10.1186/1471-2229-10-120 (PMC3017792; doi:10.1186/1471-2229-10-120)
Supplement: Additional file 1 — Unknown metabolite response ratios of the different treatments vs. Fe-sufficient controls (+Fe). List of unknown metabolites with response ratios (level in a given treatment divided by the level in the +Fe treatment) higher than 4 and a t-test significance of P < 0.01 (indicated in bold). When the ratios were lower than 1 the inverse was taken and the sign changed. [file 1471-2229-10-120-S1.DOC]

**Supplementary Table 1**

| **BinBase Metabolite ID** | **-Fe** | **24 h** | **72 h YZ** | **72 h WZ** |
| --- | --- | --- | --- | --- |
| 199205 | **6.3** | **13.1** | **4.3** | 2.3 |
| 199242 | 2.3 | **7.5** | **7.1** | **3.1** |
| 200421 | -1.9 | 2.7 | **6.8** | -1.3 |
| 200463 | 2.7 | 2.5 | **4.8** | 1.5 |
| 200523 | 2.7 | **4.6** | 3.3 | 2.7 |
| 200624 | -3.0 | **-6.1** | -1.5 | 2.0 |
| 200844 | 2.7 | **4.8** | **5.1** | 2.7 |
| 200925 | **-7.4** | -2.0 | 1.0 | 2.2 |
| 201005 | -1.7 | **-5.9** | -2.5 | **-6.3** |
| 202178 | 3.8 | **5.8** | 3.4 | 2.1 |
| 202293 | 8.2 | **21.7** | 6.7 | 2.7 |
| 202808 | 2.6 | **6.7** | 3.5 | 2.1 |
| 203241 | 1.4 | 1.7 | **4.0** | 2.2 |
| 207227 | **4.2** | **6.7** | **6.5** | 2.3 |
| 208770 | 1.7 | 2.6 | **4.0** | 4.0 |
| 211890 | 1.8 | 3.9 | **7.6** | 2.6 |
| 211891 | **17.6** | **145.9** | **69.5** | 6.3 |
| 211991 | **32.5** | **5.1** | **4.4** | 2.0 |
| 212373 | 4.8 | **7.9** | 2.0 | 2.4 |
| 212672 | **37.5** | 7.7 | 3.1 | 1.7 |
| 217840 | **-8.1** | **-4.1** | -1.2 | -1.4 |
| 217841 | 1.1 | 1.3 | **4.5** | 1.8 |
| 218550 | 1.4 | 2.2 | **4.2** | **4.1** |
| 218596 | 1.4 | 2.1 | **4.6** | 4.6 |
| 218761 | 3.4 | **6.7** | 3.9 | 1.9 |
| 218766 | 1.2 | 2.4 | **4.9** | 2.0 |
| 218767 | 1.6 | 3.9 | **7.0** | 2.8 |
| 218769 | 4.5 | **41.7** | **5.0** | 6.0 |
| 218771 | **6.6** | **6.6** | **6.2** | 2.1 |
| 218773 | 1.2 | **4.0** | **8.3** | **4.1** |
| 218798 | 1.5 | **10.1** | 8.3 | 4.0 |
| 218824 | 2.1 | -1.7 | 4.8 | **11.3** |
| 218829 | 3.1 | 3.3 | 2.8 | **6.1** |
| 218832 | 1.3 | -1.5 | -1.2 | **4.1** |
| 218834 | 3.1 | 3.8 | **9.7** | **5.1** |
| 218847 | 1.4 | 2.3 | **4.2** | 4.3 |
| 218852 | 2.7 | 3.6 | **9.7** | **5.4** |
| 218867 | **4.9** | **8.7** | **10.0** | **9.1** |
| 218986 | -3.6 | **-5.3** | -1.2 | 1.3 |
| 218993 | **-4.1** | **-6.9** | -1.4 | -1.3 |
| 218995 | -1.1 | 1.8 | **4.1** | 2.3 |
| 219171 | **5.1** | **9.3** | **4.9** | 1.8 |
| 219181 | 2.0 | 2.1 | **4.3** | 1.5 |
| 219185 | 3.2 | **5.4** | 3.1 | 1.3 |
| 219513 | 4.2 | **11.0** | 3.8 | 2.1 |
| 220143 | 1.0 | 1.7 | **4.5** | 1.7 |
