# Supplementary figures and images for: Changes in the proteomic and metabolic profiles of Beta vulgaris root tips in response to iron deficiency and resupply
Source: BMC Plant Biol. 2010 Jun 21;10:120. doi: 10.1186/1471-2229-10-120 (PMC3017792; doi:10.1186/1471-2229-10-120)

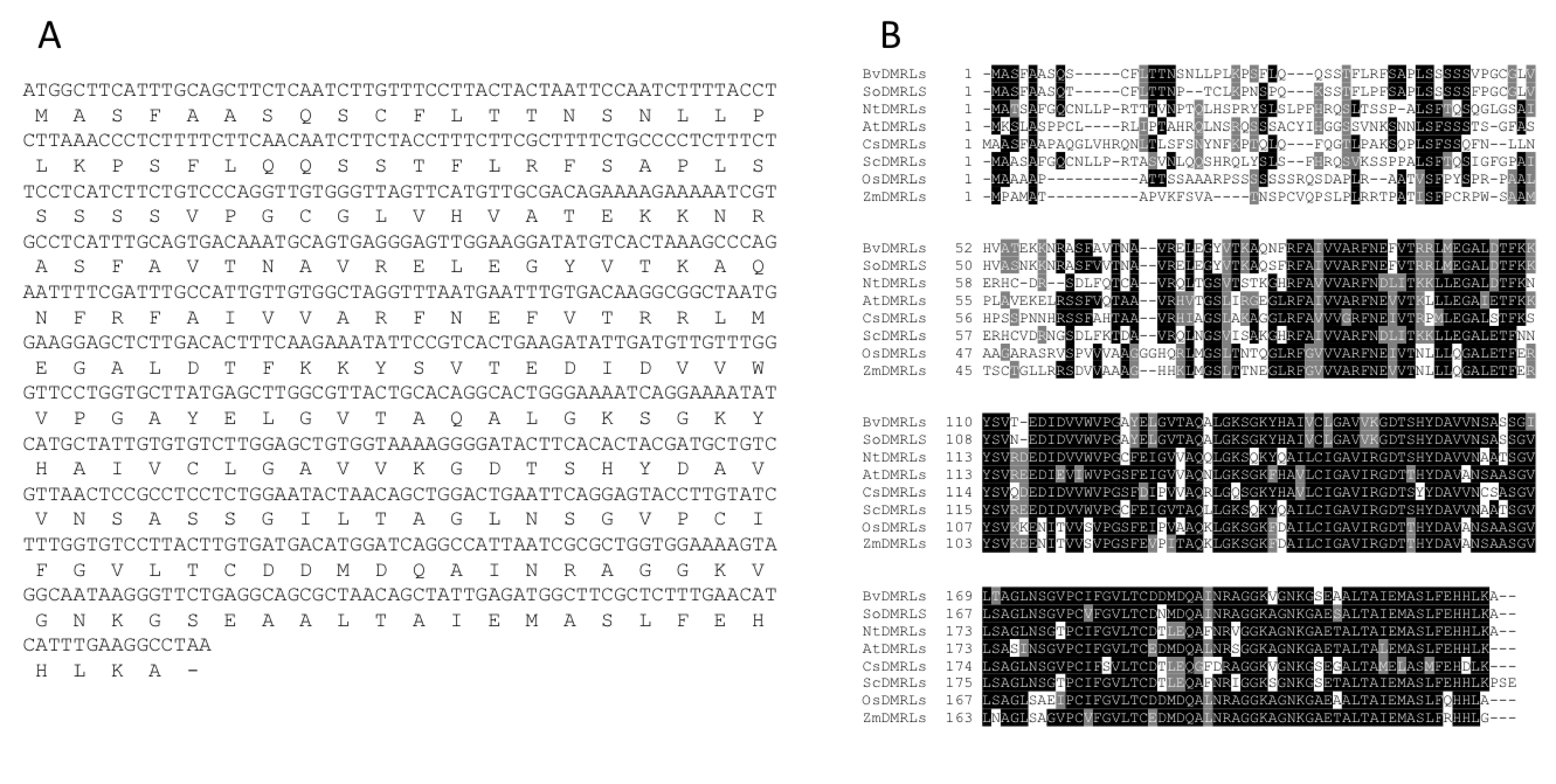

Supplement: Additional file 2 — DMRL synthase sequence and alignment. DMRL synthase sequence (A) in Beta vulgaris (GQ375163) and alignment (B) of DMRL synthase protein from Beta vulgaris (Bv) and Spinacia oleracea (So; AAD44808.1), Nicotiana tabacum (Nt; AAQ04061.1), Arabidopsis thaliana (At; AAD44810.1), Cucumis sativus (Cs; ABZ88150.1), Solanum chacoense (Sc; ACB32230.1) and Oryza sativa (Os; ACS94980.1) and Zea mays (Zm; ACG35456.1). More information about the DMRL sequences can be found at http://www.ncbi.nlm.nih.gov/protein/ [file 1471-2229-10-120-S2.TIFF]
